# Growth Dependent Changes in Pressure Sensing Walkway Data for Turkeys

**DOI:** 10.3389/fvets.2018.00241

**Published:** 2018-10-09

**Authors:** Jody A. Kremer, Cara I. Robison, Darrin M. Karcher

**Affiliations:** ^1^Department of Animal Science, Michigan State University, East Lansing, MI, United States; ^2^Department of Animal Sciences, Purdue University, West Lafayette, IN, United States

**Keywords:** turkey, gait, lameness, force, locomotion

## Abstract

Genetic selection for rapidly growing turkeys has created an unfavorable consequence impacting the skeletal system resulting in long bone distortions. These distortions have resulted in locomotor problems, gait abnormalities, leg weakness, or lameness issues. These effects raise welfare concerns along with animal agriculture inefficiency in the form of lost product. The purpose was to determine baseline gait and force distribution in visibly unimpaired growing turkey hens. Hendrix commercial turkey hen poults (*n* = 100) were placed on pine wood shavings providing 0.78 m^2^ per bird with *ad libitum* access to feed and water at the MSU Poultry Farm. Fifty hens were randomly selected at 5 weeks and identified with a leg band to ensure longitudinal data collection. The turkeys were walked across a pressure-sensing walkway (PSW, Tekscan, Boston, MA) and weighed at 5, 6, 8, and 10 weeks of age. PSW collected data on gait length, gait time, step force and step length, and the statistics were analyzed with SAS. Both temporospatial data, including step time and step length, and kinetic data, including peak downward force, and vertical impulse, were recorded. Body weight increased linearly with age (*P* < 0.001), demonstrating a typical growth pattern. Gait cycle time and peak vertical force (PVF) all displayed no difference between right and left sides, indicating that the hens had no detectable gait abnormalities. Gait velocity increased with age (*P* = 0.02) suggesting hens' growth impacted their gait velocity. The gait cycle time (*P* < 0.01) did not correspond with age. PVF increased linearly with age (*P* < 0.01) from 6 weeks (2.23 kg) to 10 weeks of age (5.91 kg). PVF/kg body weight (*P* < 0.01) increased from 6 weeks of age (96.9% BW) to 8 weeks of age (106%BW). Overall, the birds were not lame and some data was influenced by the hen's adjustment to the materials or stage of growth; in contrast, some temporospatial data did not coincide with age. The PSW could be used to detect locomotor issues in commercially produced turkey hens providing another tool for assessing well-being.

## Introduction

Given the increasing human population, animal agriculture must adapt by producing more product with the same amount of resources. Livestock industries overall have greatly improved their efficiency in the past several decades. The main contributor to their success has been genetic selection for the desired traits. Artificial insemination and an increase in genetic testing capabilities have been crucial in carrying out the selective breeding for genetic improvement. Commercial turkey species have been selected for rapid growth and increased feed efficiency (1). Consequently, an increasing number of leg problems, specifically long bone distortion, spiral fracturing, and tibial dyschondroplasia in commercial turkeys have occurred because of these expedited changes in growth rate and increased breast muscle (2). Turkeys are not the only species susceptible to these challenges; similar concerns have occurred in broiler chickens (3). As a result, commercial producers face welfare issues as well as an economic dilemma with these long bone deformations.

Bipedal animals exhibit unique gait characteristic in contrast to quadrupeds. Bipeds have mass farther from the ground, so they require more balance than quadrupeds. The most common classes of bipedal species are humans, non-human primates, and avians. A pressure-sensing walkway (PSW) provides a non-invasive analysis and diagnostic tool for identifying locomotor impairment. Typically, PSW have been used in human research, specifically, to identify foot pathologies by looking for plantar pressure asymmetry (4). Pressure-sensing walkways identify numerous components of temporospatial and kinetic data and can be helpful in determining variables leading to locomotor problems. Recently, PSW have been used to compare the gait parameters of male turkeys from four different strains throughout growth and assess the effects of gait on bone development (5). A PSW has been used in conjunction with tri-axial accelerometers to quantify the landing force of hens jumping off perches of differing heights (6) and sheep of different age groups have been evaluated to determine the effects of aging on kinetic parameters (7). However, this technology is relatively new and not widely used in animal production; therefore, obtaining gait characteristic data from sound turkeys is imperative.

Gait analysis has been done successfully with Pekin ducks 14 days and older using a 3-point rubric in addition to a PSW (8). The evaluation of these ducks found no difference at 14 days but observed lameness as they got older. However, the relationship between management decisions, age and weight are unclear, but the increasing age and weight were positively correlated with lameness. Oviedo-Rondón et al. (5) reported tom turkeys gait was associated with increasing leg deformations and increasing age. Decreased gait velocity and increased force as a percentage of body weight were observed with increasing age as well. Correcting the bone density deficiencies caused by genetic selection for rapid growth must start with baseline data of what is normally seen in differing species, breeds, and sexes of poultry.

The purpose of this study was to determine normal baseline gait data for growing turkeys. There have been a few papers with extensive detail describing bird gait and the impact on the gait parameters discussed below (9, 10); however limited research has been conducted using PSW on avian species. Establishing normal gait data will provide information allowing for comparisons to data collected in research and commercial settings.

## Materials and methods

### Ethics statement

This study was carried out in accordance with the recommendations and approval of the Michigan State University Institutional Animal Use and Care Committee.

### Housing and data collection

One hundred female Hybrid strain turkeys were raised at the Michigan State University Poultry Teaching and Research Center. They were floor reared in a barn 6.7 × 11.6 m. The turkeys had free choice access to feed and water. Turkeys were fed according to the Hybrid Converter management guide. At 5 weeks of age fifty poults were randomly selected for participation in the project; these turkeys were given a leg band to identify them in addition to a green marking on their back with livestock paint. Turkeys were walked over the PSW on week 5, to allow them to acclimate to the setup. Body weight and pressure-sensitive walkway (PSW: Tekscan Inc., South Boston, MA) data was collected at 6, 8, and 10 weeks of age.

The PSW is designed for measuring gait parameters of animals and humans. The PSW does so by recording multiple foot strikes as a real time movie that can assess the differences and similarities between several foot strikes. The PSW used in this study was 0.9 m long and 0.6 m wide with a total of 4,576 sensels resulting in 1.4 sensels/cm^2^. The walkway sensors were calibrated and equilibrated each day according to the manufacturer's specifications using an animal phantom with 11 kg of mass. Each sensor was calibrated in kPa using the Walkway software to ensure that both pressure sensors were working correctly. Then, a previously created calibration file, specific for turkeys was uploaded to the PSW (11).

**Figure 1:**
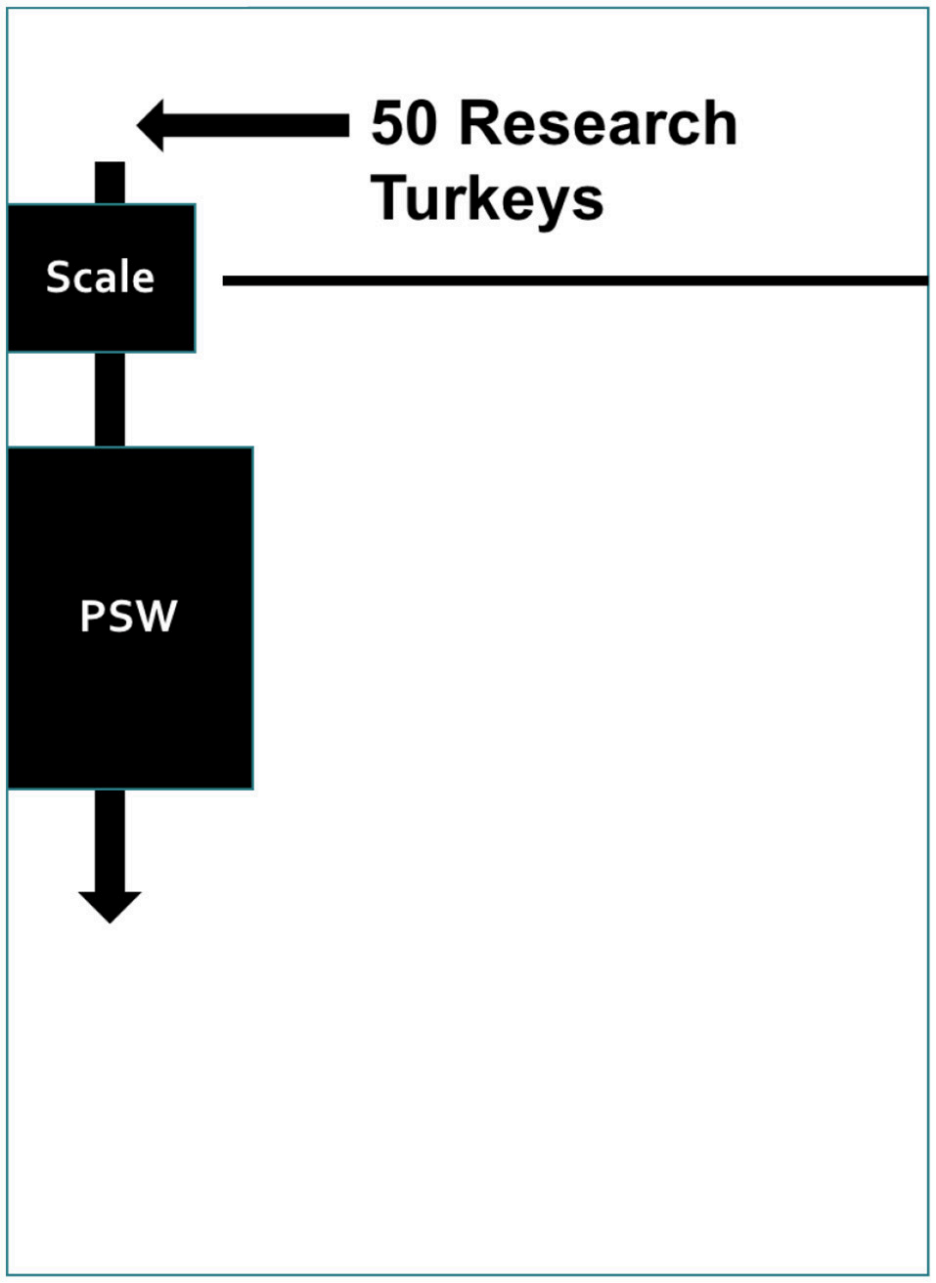
A diagram of how the experimental pens were set up on sampling days. The 50 research turkeys were gathered behind temporary panels in the back of the barn. During sample collection each turkey was individually guided onto the scale, weighed, and then walked over the pressure sensing walkway.

On sampling days, mobile panels were placed ~2.5 m from the back wall of the barn and used to separate the research turkeys from the remainder of the flock. A small gap remained in this temporary pen where the scale was placed (Figure 1). The PSW was placed alongside a wall to impel the turkeys to walk in a straight line.

Following set-up, the fifty research turkeys were gathered into the temporary pen at the back of the barn. Then, each turkey was guided onto the scale, weighed, and walked across the PSW. One researcher would encourage the hen to walk along the PSW and, if possible, turn the hen around to walk across a second time to obtain more strides The PSW files were recorded and saved using dedicated software (Walkway 7.0; Tekscan Inc., South Boston, MA).

**Figure 2:**
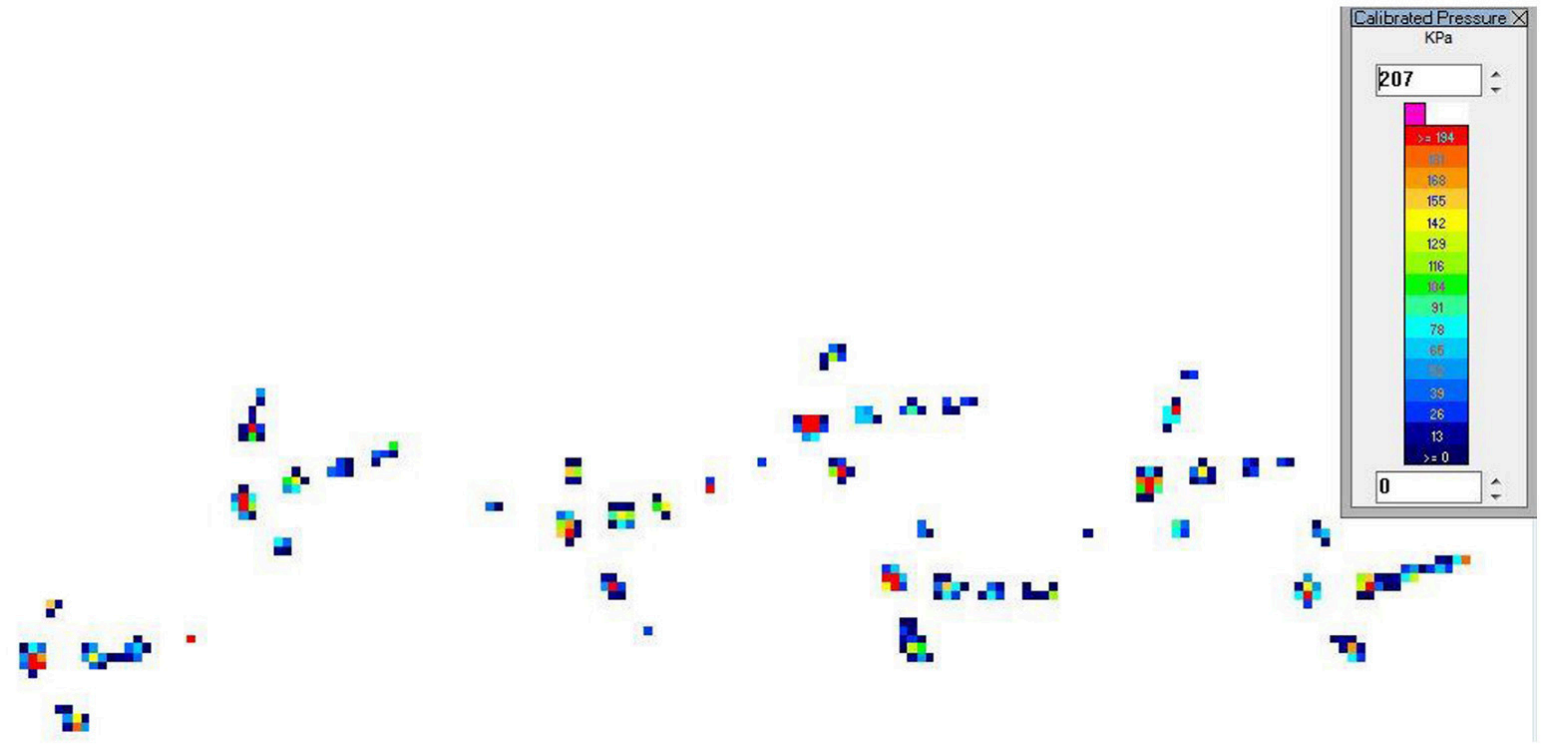
Images collected from turkeys walking over the pressure sensing walkway. Footprints appear in different colors with each color representing different amounts of pressure.

The PSW files were reviewed using Walkway software and for a walk to be considered valid, it needed to contain at least four continuous strikes, or complete foot prints, and the walking pattern had to be linear (Figure 2). The average number of strikes on valid files was 7. Right and left foot strikes were manually designated to allow the software to analyze the right and left sides in addition to the difference between them. Peak vertical force (PVF) and vertical impulse were determined. Both variables were normalized to the turkey's body weight and presented as percentages of body weight (%BW). Limb duty factor was estimated as total contact time divided by gait cycle duration (12, 13). A description of the gait variables can be found in Table 1.

**Table 1:** Gait parameters and definitions.

**Parameter**	**Definition**
Cadence (steps/min)	Or step frequency, the number of steps taken per minute
Gait velocity (m/s)	Or speed, calculated by dividing the distance the bird traveled across the walkway divided by the time interval from first to last contact
Contact time (s)	Amount of time the foot was in contact with the sensor
Single support time (s)	Amount of time the foot is in contact with the sensor while the opposing foot is not in contact with the sensor
Step length (cm)	Distance between most posterior contact points on subsequent footfalls of opposite feet
Stride length (cm)	Distance between posterior contact points of two subsequent footfalls of the same foot
Gait cycle time (s)	Average time from first contact of the foot to subsequent first contact of the same foot
Duty factor	Segment of the total stride during which foot was in contact with the walkway

### Statistical analysis

Statistics were analyzed using the MIXED procedure of SAS 9.3 with age and bird as class variables and bird as random. Least squared means were calculated as well as linear and quadratic contrasts by age. Results are presented as LSMeans ± SEM. Significance was considered at *P* < 0.05.

## Results

Each week data from the same 50 turkeys were collected; however, not all birds performed a valid walk. At 6 weeks of age 29 turkeys performed valid walks with 36 valid walks at 8 weeks of age and 22 valid walks at 10 weeks of age. The weight of the turkeys increased linearly from 6 to 10 weeks of age (*P* < 0.01) documenting proper growth and healthy hens, although body weights were around 5–7% below the performance objectives (14). Accordingly, PVF increased linearly with age with week 10 having a force of 57.9 N (*P* < 0.01). When PVF was adjusted to account for kg BW, the resulting calculation was close to 100%; however, with a trend for a linear increase as the turkeys aged (*P* = 0.08).

Gait velocity, step length, and stride length, increased linearly as the turkeys aged (*P* < 0.01; Table 2). Cadence changed amongst the three time points, but no difference was observed (*P* = 0.22). Contact time tended to change amongst the ages (*P* = 0.052). Single support time decreased linearly with age (*P* < 0.01) with week 10 having the shortest time of 0.34 s (*P* < 0.01). Gait cycle time decreased with age with 10 week old hens having the slowest, 1.34 s, gait cycle time (*P* = 0.02). No difference in stride length, step velocity, duty factor, and peak vertical force, between the right verses left leg was observed (*P* > 0.31; Table 3).

**Table 2:** Number of turkey hens, average body weight, and gait parameters collected at three different ages via the pressure sensing walkway.

	**6 Weeks**	**8 Weeks**	**10 Weeks**	**Age effect *P*-Value**	**Linear contrast *P*-value**
n	29	36	22		
Weight (kg)	2.32 ± 0.042^a^	3.99 ± 0.04^b^	5.80 ± 0.05^c^	< 0.01	< 0.01
Cadence (steps/min)	78.9 ± 4.4	70.7 ± 3.9	81.1 ± 5.1	0.22	0.75
Gait velocity (m/s)	0.18 ± 0.02^a^	0.20 ± 0.01^a^	0.26 ± 0.02^b^	0.02	< 0.01
Peak vertical force (*N*)	21.9 ± 0.7^a^	41.2 ± 0.7^b^	57.9 ± 0.8^c^	< 0.01	< 0.01
Peak vertical force (%BW)	96.9 ± 1.3^a^	106 ± 1.2^b^	101 ± 1.5^a^^c^	< 0.01	0.08
Vertical impulse (%BW)	68.3 ± 4^a^	81.0 ± 3.3^b^	62.2 ± 4^a^^c^	< 0.01	0.28
Contact time (s)	1.10 ± 0.07	1.28 ± 0.06	1.04 ± 0.08	0.05	0.59
Single support time (s)	0.54 ± 0.02^b^	0.41 ± 0.02^b^	0.34 ± 0.02^c^	< 0.01	< 0.01
Step length (cm)	13.7 ± 0.60^a^	16.4 ± 0.54^b^	17.9 ± 0.69^b^^c^	< 0.01	< 0.01
Stride length (cm)	26.5 ± 1.0	31.9 ± 0.9	35.7 ± 1.1	< 0.01	< 0.01
Gait cycle time (s)	1.69 ± 0.08^a^	1.66 ± 0.08^a^	1.34 ± 0.10^b^	0.02	0.01
Duty Factor (Right Leg)	0.65 ± 0.03^a^	0.78 ± 0.03^b^	0.79 ± 0.03^b^	< 0.01	< 0.01
Duty Factor (Left Leg)	0.66 ± 0.02^a^	0.78 ± 0.02^b^	0.70 ± 0.02^a^	< 0.01	0.15

abc *Differing letters within a horizontal row are different P < 0.05*.

**Table 3:** Differences between the right and left side data for different gait parameters obtained via the pressure sensing walkway.

	**6 Weeks**	**8 Weeks**	**10 Weeks**	***P*-Value**
Peak Vertical Force (*N*)	0.32 ± 0.6^1^	0.16 ± 0.5	−0.95 ± 0.7	0.31
Stride length (cm)	−1.58 ± 0.8	−0.67 ± 0.7	−0.29 ± 0.9	0.47
Impulse (kg/sec)	−0.04 ± 0.12	−0.07 ± 0.10	−0.20 ± 0.13	0.65
Step velocity (m/s)	−0.01 ± 0.01	0.00 ± 0.01	−0.02 ± 0.02	0.67
Duty Factor	−0.008 ± 0.02	−0.007 ± 0.03	0.088 ± 0.05	0.40
Gait cycle time (s)	−0.00 ± 0.04	0.00 ± 0.04	−0.19 ± 0.11	0.06

1 *Negative numbers indicate the left-side data was greater than the right-side data*.

## Discussion

The majority of the animal studies have been conducted using quadruped companion or farm animals (7, 15–17). However, the gait analysis of tom turkeys, Pekin ducks, and broiler chickens have been assessed using a PSW (5, 18, 19). The turkeys used in this study had no apparent leg defects and were not observed to have any gait abnormalities.

Using the PSW with species that habitually form a flock presents challenges; stress induced by animal isolation has been observed when sheep were prompted to walk alone over a PSW (20). The stress on the animal inevitably creates an abnormal walking pattern that would not be exhibited in the familiarity of a flock. Occasionally, a turkey would bolt off of the PSW on their first walk or refuse to walk upon encountering the PSW. This data was declared invalid due to not reflecting a typical walking pattern. In future studies, additional pre-training, and acclimation to the walkway or setting up the walkway outside of their housing environment may be advantageous.

Birds were allowed to walk freely across the walkway; however, if the bird paused or stopped then researchers encouraged it to continue moving. The goal was to have the turkeys walk as naturally as possible over the walkway. The gait velocity increased linearly with age from 0.18 m/s at 6 weeks to 0.26 m/s at 10 weeks. This gait velocity was ~40% slower than velocities reported (5) at similar ages. The speeds in the current study are similar to low speeds reported (19) in Brown Leghorns walking across a force plate. The turkey hens might have been hesitant to walk on the walkway, thereby reducing their normal walking speed. The current study found no variation in cadence (Table 2) between the 3 bird ages so although speed increased with age the number of steps taken per minute remained constant. Cadence and step length can be influenced by speed (9) with birds choosing to increase step frequency instead of step length. In the current study, step length increased suggesting that changes in velocity may be due to growth and changes in leg length.

The PVF increased with age (*P* < 0.01; Table 2). This was anticipated as the increase in force is a main contributor to the locomotor problems observed in the industry. The hen's body weight directly correlates to bird age and concluding the study at 10 weeks limited the potential for locomotor problems to develop with increased body weight. These findings are compatible with a previous turkey gait analysis study finding even higher PVF data for older and presumably heavier tom turkeys (5). PVF between two duck breeds, the Pekin, selected for higher breast muscle mass, and the Mallard, no selection pressure, found Pekin ducks had a greater PVF as a percentage of body weight compared to Mallards (21). The observed vertical impulse was not consistently increasing or decreasing over the three data collections. No change in vertical impulse was observed in the studies on chickens and ducks either (21). This researcher described it as being expected because of the balancing factors of an increase in double foot support time and PVF.

The contact time tended to vary between weeks, with 8-week old turkeys tending to have an increased contact time compared to the other weeks. At 6 and 8 weeks, gait cycle time was much longer suggesting the birds may have paused or been more hesitant during data collection. The inconsistency in contact time with age was reported in previous gait analyses (5). This can be explained by the variability in the turkey's pace of a single walk. Step length increased linearly with age from 13.7 to 17.9 cm, which is a logical observation due to an increase in overall body size. However, in a comparable study this trend was not detected (5) and an average step length of 26 cm was reported across all ages of birds. This contradictory data may be related to the increased gait velocity observed in this study but not the other. The body confirmation and weight distribution change is dramatic as the domestic turkey grows which could alter the gait kinematics.

When velocity remains relatively constant duty factor is calculated: contact time/gait cycle time with a duty factor >0.5 indicating a support phase or the fraction of a stride in which the foot is in contact with the ground (22, 23). In the current study, duty factor did increase linearly from 0.65 to 0.79 as the birds aged in the right leg (*P* = 0.02). The average duty factor in 8 weeks turkey hens was 0.78, which is similar to that reported in 47 days old tom turkeys (5). In tom turkeys both velocity and duty factor decreased with age, whereas the current study found the opposite response. The turkey hens in the current study weighed, on average, 4 kg at 10 weeks while the turkey toms weighed 7.33 kg at 47 days (5) so perhaps breast size and body position while walking contribute to the differences between the results. Daley and Birn-Jeffery found that due to their crouched posture many galliform species have a shorter stance time and lower duty factor in comparison to other avian species of similar size (23). A recent publication examined kinematic gait differences between wild and domestic turkeys and reported that duty factor did not decrease with speed in either wild or domestic turkeys (24). In the current study, speed increases linearly from 0.18 to 0.26 m/s; however, duty factor does not decrease as has been reported in other avian gait research (5, 12, 23). A 0.08 m/s increase in velocity while statistically significant may not be biomechanically relevant. The PSW is quite sensitive and can likely detect minute changes in gait that are unperceivable to the naked eye.

The difference in gait data between the right and left sides was necessary to evaluate the soundness of the turkeys. Without differences in PVF (*P* = 0.31), the weight distribution was even between left and right sides. This verifies that the turkeys were structurally sound and baseline data is valid. Additionally, stride length, step velocity, and duty factor were not different between right and left legs. This lack of significance is beneficial because it indicates the turkeys were not favoring one side over the other, which would have suggested an abnormal, lame gait pattern. Further research would need to be conducted to determine if limb dominance exists in growing turkeys and how to distinguish those gait patterns from birds with lameness problems.

Overall, the data generated from this study provides the information on baseline gait data values for commercial turkey hens from 6 to 10 weeks of age.

## Author contributions

DK and CR designed the project and made edits to the manuscript. CR assisted in methods and analysis of data collected. JK collected data, analyzed the data, and drafted the manuscript.

### Conflict of interest statement

The authors declare that the research was conducted in the absence of any commercial or financial relationships that could be construed as a potential conflict of interest.
